# Local people’s perception of the impacts and importance of ecotourism in Central Nepal

**DOI:** 10.1371/journal.pone.0268637

**Published:** 2022-05-27

**Authors:** Suraj Upadhaya, Sarada Tiwari, Beeju Poudyal, Sagar Godar Chhetri, Nabin Dhungana

**Affiliations:** 1 Department of Natural Resource Ecology and Management, Iowa State University, Ames, IA, United States of America; 2 Himalayan Conservation and Research Institute Nepal, Kathmandu, Nepal; 3 Tribhuvan University, Institute of Forestry, Pokhara, Nepal; 4 Faculty of Forestry, Agriculture and Forestry University, Hetauda, Makawanpur, Nepal; 5 College of Forestry, Agriculture & Natural Resources, University of Arkansas, Monticello, AR, United States of America; 6 Department of Natural Resources and Environmental Studies, College of Environmental Studies, National Dong Hwa University, Hualien, Taiwan; 7 Natural Resources Conservation Nepal (NRCN), Kathmandu, Nepal; Northeastern University (Shenyang China), CHINA

## Abstract

Ecotourism contributes to conserving natural resources and promoting natural and cultural resources stewardship. However, without the strong support and involvement of local people, it is not easy to achieve the stated goals. This study aimed to understand the local people’s perception of the impacts and importance of ecotourism. We conducted a semi-structured interview of 167 respondents from Chitwan National Park (CNP), Nepal’s prime center for ecotourism. The result revealed that more than 70% of respondents are interested in ecotourism activities, and their interests are significantly affected by their age, academic qualification, and occupation. Local people from the study area perceived that infrastructure development and increase in the knowledge base are significant impacts of ecotourism. The study showed that local people’s socioeconomic and demographic characteristics significantly influenced their perceived impacts of ecotourism. Detailed understanding and consideration of socioeconomic and demographic characteristics can contribute to effective outreach and planning process, potentially resulting in the higher promotion of ecotourism.

## 1 Introduction

The tourism industry is one of the world’s largest industries and is associated with many sectors of the world’s economy. It creates jobs, drives exports, and generates prosperity across the globe [1,2]. Tourism contributes 10.4% of total gross domestic products (GDP), approximately US $8.8 trillion annually, creating 319 million jobs to the world economy [1]. In Nepal, travel and tourism contributed 7.9% of total GDP, approximately US $746.3 million annually, and created 1.05 million jobs [1].

Ecotourism is defined as "responsible travel to natural areas that conserve the environment, sustains the well-being of the local people, creates knowledge and understanding through interpretation and education of all involved; visitors, staff and the visited" [3]. It represents nature-based travel to a relatively undisturbed touristic destination [4]. In both developed and developing countries, ecotourism is viewed as an engine of economic advancement and a pathway for improving the livelihoods of communities that might otherwise struggle to grow and flourish [5,6]. Ecotourism helps poverty alleviation, job creation, income redistribution, and export of domestic products to international markets [7,8]. Ecotourism can promote sustainable development through managing biological diversity and ecosystem services and functions by ensuring quality tourism experiences and absorbing or adapting to the pressures of tourists [9]. It focuses on community development, poverty alleviation, biodiversity conservation, and traditional economic activity, including agriculture, livestock, and hunting [10]. In developing countries like Nepal, it supports employment, markets, and cultural conservation and promotes ownership, management, and equitable sharing of benefits from natural resources among communities in the local area [11–13].

Nepal is popular for natural, historical, cultural, and adventure activities. For international tourists, it is famous for its low-cost tourism destinations. Due to higher buying power resulting from a favorable currency exchange rate and lower living expenditures, inbound tourist numbers are high [14]. The majority of the tourists prefer Nepal as a destination for trekking, rafting, wildlife safari, and cultural pilgrimage. Nepal’s rich biodiversity is also another high attraction for tourists. This includes watching spectacular species such as the One-horned Rhino, Asian Elephant, and Royal Bengal Tiger. Many ecotourists opt for their destination in the protected areas of Nepal, such as Chitwan National Park (CNP), Bardiya National Park, and Suklaphanta National Park. While natural attraction provides the foundation of tourism in any country, promoting an increase in tourist numbers depends on positive tourism experiences [15].

Similarly, the availability of facilities, quality delivery of services, and hospitality help sustain the quality and growth of tourism [15]. CNP has been serving these facilities through local people, entrepreneurs, and people in business. The primary services provided by the local people are manufacturing handmade souvenirs, showcasing their tradition through dance, homestay, and guiding the ecotourists. In turn, the local community earns foreign currency, advances their livelihood, and is motivated to develop ecotourism.

Several studies have explored the perceived impacts of ecotourism on the local community. Developing ecotourism industries and interactions with tourists significantly impact local communities [16]. These interacting forces can influence the communities’ values, behavior, lifestyles, and quality of life [17]. The development of ecotourism industries can positively and negatively affect both the natural resources and local communities’ livelihoods [15]. It is also found that ecotourism has supported the conservation of natural resources, promoted alternative energy sources, and enhanced women’s empowerment [12]. Households involved in ecotourism-related activities have a significantly higher living standard [18]. Their purchasing power will be greater than their counterparts and vice-versa [19]. There is a trade-off between economic benefits and environmental and socio-cultural costs that requires a balance between the protection of natural resources, livelihood improvement, and community development [20].

People’s perceptions of the sustainability of ecotourism revealed different perspectives. The perspectives of the individuals are influenced by their socio-demographic factors. For instance, the demands and necessities of the low-income and high-income individuals are different. As a result, they observe the resource from their perspectives. Low-income residents benefited from fuelwood, non-timber forest products, and fodder [21].

In contrast, high-income individuals benefited from timber extraction, forest-based enterprises, and large businesses. These benefits motivate local people to conserve forestlands and associated biodiversity. Also, the provision of wildlife damage compensation schemes provided by the Nepal Government has dramatically improved the attitudes of local people toward conservation [22]. Similarly, the revenue generated from ecotourism can be used to manage and conserve wildlife species and their habitat [23], community development, conservation education, and skills development [24]. Besides, ecotourism promotes and keeps alive local culture and traditions. Many hotel owners encourage tourists to visit Tharu (ethnic community) villages in CNP to understand better Tharu’s culture, art, living style, and traditions [25]. The community shows a warm welcome and respect to the tourists. The tourist visits the area to see the local culture, encouraging locals to conserve their indigenous practices. Also, local people perceived that the presence of tourists enhanced their pride in the region [26]. Ecotourism also played a crucial role in sustaining traditional practices and making prosperous communities.

Though ecotourism has many positive aspects, there are also some negatives. The fast growth of the tourism industry might have negatively impacted society, the environment, and the economy [27]. Drug abuse, illegal sexual activities, and other illegal activities have been enhanced due to tourism activities [25]. Also, a report by [28] found that the consumption of drugs and alcohol, robbery, and prostitution has increased in this area. These negative economic externalities encompass a rise in the price of goods and services [29]. Due to Nepal’s small and tourism-dependent economy, the pricing effects on commodities might become a permanent and nationwide phenomenon that affects the lives and welfare of all inhabitants [30]. In addition, tourism might have negative impacts on forests and biodiversity. Unplanned tourism activity is responsible for environmental degradation, waste generation, and pollution [13,31]. Therefore, unmanaged tourism development activities are a risk to the sustainability of the tourism industry.

In sum, previous research has identified several impacts of ecotourism, even when ecotourism helps uplift people’s livelihood. However, those studies focused on the major tourist flow areas where there are sophisticated services provided by hotels, lodges, and big business owners to the visitors. Also, rural and urban residents’ perception of ecotourism is significantly different [32]. So, to our knowledge, there is a paucity of information on local peoples’ perception of ecotourism in the study area. Also, its impacts on various social and economic aspects are still lacking.

A previous study also suggested that a host community’s participation in ecotourism development is closely linked to its access to knowledge about ecotourism and its related ability to control and manage local tourism resources [33]. Another study argued that understanding whether the host community benefits from ecotourism activities is crucial for sustainable management [34]. Host community participation facilitates access to benefits from ecotourism activities, but it also entails the right to a say in exerting controls on the ecotourism development process [6]. A sense of ownership and perceptions of ecotourism by host communities is an important issue for ecotourism development [6]. Today, policymakers pay increasing attention to economic development and growth and less attention to the effects of this growth on the environment, leading to the degradation of biodiversity. It is found that behavioral and functional activities in the area influenced tourism activities. It is not easy to accurately identify tourism’s social, ecological, and economic impacts [32]. Therefore, this study seeks to understand the local community’s perceptions of ecotourism impacts.

## 2 Materials and methods

### 2.1 Study area

**Fig 1:**
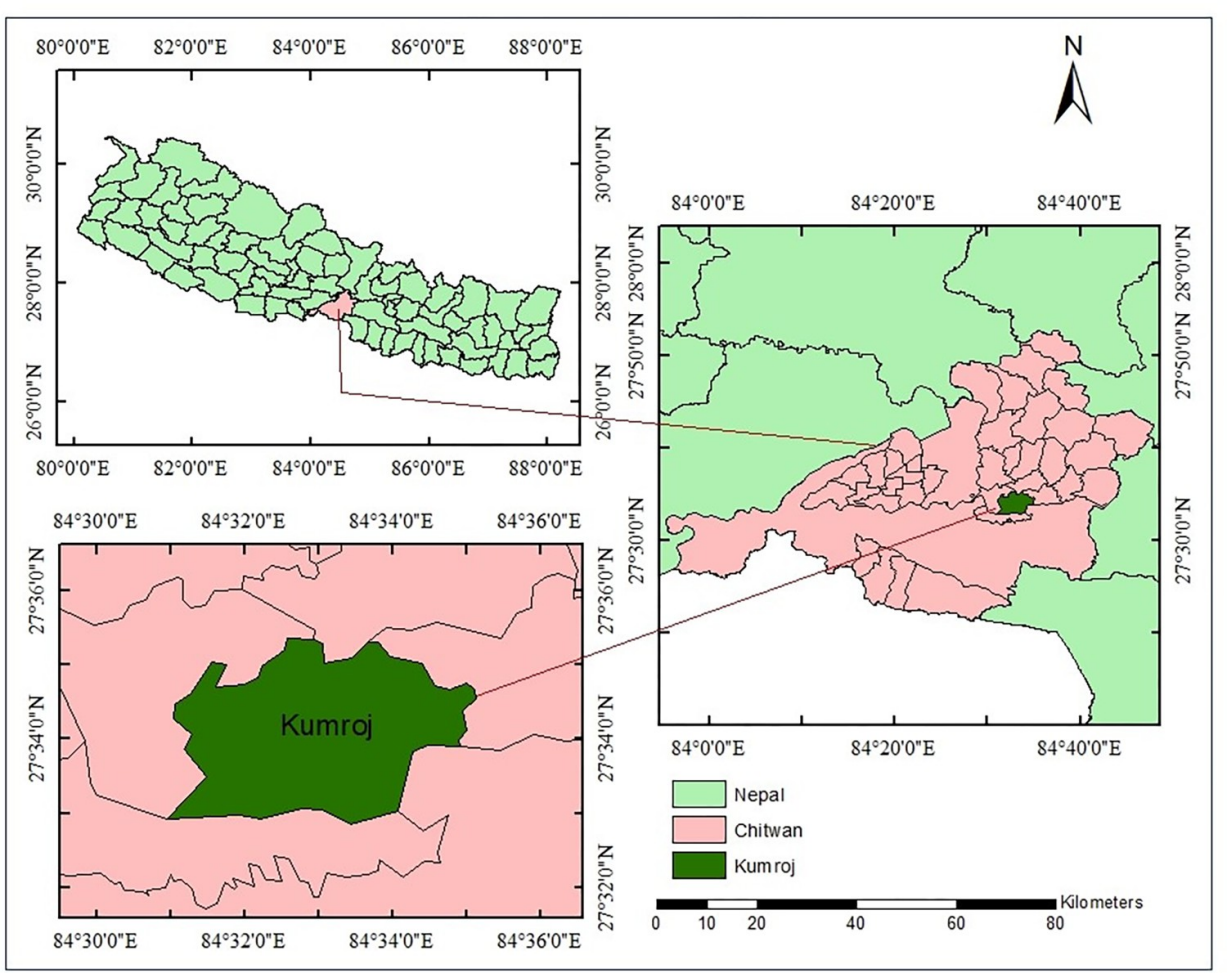
The location of the Chitwan district within Nepal and the Kumroj buffer zone within the Chitwan district.

Chitwan National Park (CNP) was the first national park declared in Nepal (in 1973) and has an area of 932 km^2^. CNP was designated as a UNESCO World Heritage Site in 1984. The Kumroj, a village and one of the potential destinations for ecotourism, covers 21.22 km^2^ of land in the buffer zone of CNP (Fig 1). The total area of Kumroj comprises 3.10 km^2^ of forest area, 12.98 km^2^ of agricultural land, 3.66 km^2^ of grassland, 0.94 km^2^ of water bodies, and the remaining 0.54 km^2^ of other lands [35]. It consists of 1,750 households with 8,082 residents representing 16 different castes comprising indigenous Tharu and Darai communities along with hill migrants [35,36]. People of Kumroj depend on agriculture as the main source of income, besides which they rely on forest resources through plantation and regeneration of forest to sustain their livelihood. The degraded forest was recovered through community participation after 1980, and ecotourism was started in 1997 [36]. The renovation of the community forest and the increased presence of wildlife in the forest managed by the Kumroj buffer zone community forests users’ group have assisted the ecosystem regeneration. Then after, they promoted ecotourism, which can support local livelihood and conservation of forest resources [37,38]. Through ecotourism activities which started in 1997, this community of forest users group is generating approx. $45,000 annually [36]. Kumroj area has prime value for recreation, cultural richness, and wilderness, making it potential for ecotourism.

### 2.2 Data collections

The target population for this study consisted of local communities living in the adjacent villages (Khumroj) of Chitwan National Park. A total of 167 households (10% of the total households) were interviewed, which is enough for generalizing the results in the study area at a 95% confidence level with a 5% margin of error [39]. The authors themselves carried out the interviews in June-July of 2019 for this study. Stratified random sampling (SRS) with a sample interval of ten was applied, i.e., every tenth household after the first randomly selected households was surveyed. The SRS method was used because it provides a well-representation of the population. The head-of-household was asked to answer the questions. Only one person had selected from one household for an interview. If the head-of-household member was not available, we interviewed a family member above 18-years-old. In addition, the head-of-household who understood the Nepali (local) language and who was willing to participate at the time of data collection was included in this study. If both cases were not possible, the household was not included in the sampling.

Face to face semi-structured questionnaire was used for data collection (S1). So far, there is no institutional review board or national ethical guidelines for social science research in Nepal. However, the study adhered to established standards in research ethics, such as keeping the personal identifier confidential and obtaining verbal consent before starting the questionnaire survey. We also maintained confidentiality by keeping the filled-up questionnaire safely, using the data only for analysis purposes, and discarded immediately after completing the data entry. Data were collected according to the convenience of respondents, and time was allocated to 25–30 minutes for each respondent. A questionnaire survey was the primary source of data collection. The four-page survey instrument included two parts. Part 1 covered the respondent’s demographic characteristics, including gender (male and female), age (recoded as two categories; Young and Old, based on the median age of Nepalese people ~ 25 years), and education (illiterate: who did not go to school or cannot read or write; literate: who went to school or can read and write). Ethnicity was measured into four possible categories. Household income was recoded into two categories (1) Less than Nepal’s median household income and (2) higher than median household income per month. The second part of the survey solicited respondents’ perceptions of various aspects of ecotourism. Respondents were asked to state their perceptions about the benefits of ecotourism. Respondents who perceived that ecotourism is beneficial answered "Yes" otherwise "No."

Similarly, those respondents who think ecotourism changed their lifestyle responded "Yes" otherwise "No." Respondents were asked about their level of interest in ecotourism and their response, and we categorized their responses into "high," "medium," and "low." These two "Yes" and "No" and "high," "medium," and "low" three answers were used in the Chi-square test. Due to space limitations and the survey’s primary objective, we did not ask about the quantitative benefits of ecotourism.

In addition to the questionnaire survey, we also conducted key informant surveys (n = 5) with personnel from Kumroj Buffer Zone Community Forest Users Group and the homestay management committee to understand the local area better. During the key informant survey, we discussed existing services available for ecotourism, the socioeconomic situation, and the operational plan of the group.

### 2.3 Data analysis

Data were reviewed and checked daily for completeness, consistency, and accuracy. After finishing the data collection, all the data were rechecked, edited, coded, categorized, entered, and analyzed in the computer program using R software.

For further analysis, we grouped variables into different categories. We described perception with seven different dependent variables., 1) Local people’s interest in ecotourism: this variable can be defined as the residents’ have an attentiveness or being attracted to continue their business; 2) local people’s perceptions about the economic impacts of ecotourism: this means whether people believed or have some evidence of raising the economic activity in their areas due to ecotourism; 3) impacts of ecotourism in changing respondents’ lifestyle: this variable identifies the resident’s perception regarding their improvement of different facilities such as switching from fuelwood to gas, more conscious about the health and safety, and being more informed; 4) impacts of ecotourism in causing economic inequality in the study area: it explains about the negativity of the inflow of tourism. For example, increase in bus fares, land, room, house, and overall market; 5) ecotourism as the reason for the increase in commodity price: the variables try to reveal the disproportion of the goods and services such as market prices of the basic daily necessities; 6) perceptions towards the impacts of ecotourism in infrastructure development in their area: this explained about whether resident’s experienced about the development of infrastructure in their community such as road construction and maintenance, electricity and other utilities; and 7) impacts of ecotourism on knowledge improvement of locals: the variable explains how the local people gather the information regarding the ecotourism activities and their level of awareness about the current ecotourism demand in the market.

Similarly, we have the socioeconomic characteristics of the respondents as independent variables. Age, gender, caste, education status, household income, and occupation were the major socioeconomic factors. We grouped ethnicity into four major groups, namely (1) Brahmin/Chhetri/Newar (BCN); (2) Janajati; (3) Madhesi; (4) Dalits. Similarly, we grouped the education level into three categories (1) illiterate, (2) school level, and 3) university level. However, for the convenience of analyzing the data, we further grouped it into two categories (1) illiterate and (2) literature. Likewise, we categorized household income into three categories, (1) low, (2) medium, and (3) high, based on the minimum salary of the Government of Nepal. Again we further group them into two categories (1) low; and (2) high using the national median income [40]. Similarly, the respondents’ ages were grouped into three categories (1) young age; (2) middle age; and (3) elderly age group. In addition, the occupation of the respondents was grouped into three major categories (1) agriculture, (2) business, and (3) others (remittance, pension, etc.). Given the nature of the data, we employed a chi-square test to assess the association of these variables to respondents’ perceptions.

## 3 Results

Of the 167 household respondents interviewed, 85 respondents were male (50.90%), and 82 were female (49.10%). The majority of the respondents were 25 years or older (Table 1). The median age of the respondent was 25 years. Most of the respondents interviewed were between the age of 15–39 years (43.71%), followed by 40–59 years (39.52%) and above 60 years (16.77%). Twenty-six percent of respondents were illiterate, while 73.66% of respondents have primary school or higher academic qualifications. The major source of livelihood was found to be agriculture. More than two-thirds of the respondents’ major source of income was agriculture, followed by business and other jobs (Table 1). The median monthly income of the respondents was NRs 14,700 (the US $1 = NRs 103). Most of the respondents (83.23%) were less than NRs 18,500 monthly income, which is the minimum salary of the Government of Nepal. Only a few respondents (6 or 3.59%) had income more than NRs. 30,000, and the remaining respondents (13.17) had income between NRs. 15,001- NRs 30,000.

**Table 1:** Socio-demographic information of respondents.

*Socio-demographic characteristics*	*Categories*	*Respondents #*	*%*
Gender	Male	85	50.90
	Female	82	49.10
Age in years	Young age (15–39)	73	43.71
	Middle age (40–59)	66	39.52
	Elderly age (60 and above)	28	16.77
Education	Illiterate (no school)	44	26.35
	School-level	108	64.67
	University-level	15	8.98
Occupation	Agriculture	136	81.44
	Business	16	9.58
	Others (governments jobs, pension, remittance)	15	8.98
Ethnicity	BCN (Bhramin/Chhetri/Newar)	106	63.47
	Janajati	42	25.15
	Madhesi	14	8.38
	Dalits	5	3.00
Household’s income (per month)	Low (Less than NRs 15,000)	139	83.23
	Medium (NRs 15,001 to NRs 30,000)	22	13.17
	High (higher than NRs 30,000)	6	3.59

### 3.1 Local people’s interest in ecotourism

The results showed that more than 70% of the respondents showed high or medium interest in ecotourism in their area (Table 2). One of the possible reasons for showing greater interest is that local people benefited from ecotourism. A chi-square test of independence was performed to examine the relationship between different socio-demographic characteristics and the respondents’ interest in ecotourism. Among six variables, an education level (p = 0.016) and occupation type (p<0.001) were statistically significant to the interest of the local people in ecotourism at a 95% level of significance (Table 2). The finding is logical because educated people were more aware of the opportunity of having ecotourism. Similarly, an occupation that depends upon the inflow of tourists in the area would increase the business’s profitability.

**Table 2:** Contingency table showing the interrelation between different socio-demographic variables and respondents’ interest in ecotourism.

Socio-demographic characteristics	Categories	Interested (count)	Not Interested (count)	Chi-squared test (p-value)
Ethnicity	BCN Janajati Madhesi Dalits	78 26 9 5	28 16 5 0	0.369
Gender	Male Female	64 54	21 28	0.094
Education	Illiterate Literate	24 94	20 29	0.016
Income	Low High	83 30	41 8	0.0127
Occupation	Agriculture Business Others	94 15 9	42 1 6	0.000
Age	<25 years >25 years	11 107	3 46	0.498

### 3.2 Perceptions of local people on the economic impacts of ecotourism

Most respondents think that ecotourism is not responsible for a change in their lifestyle. Only 9% of respondents think that ecotourism brings a difference in their lifestyle (Table 3). The study revealed that people were only fulfilling their basic needs rather than making more money which significantly improved or changed their lifestyle through tourism activities. The contingency table indicates that variables such as income (p = 0.003), age (p<0.001), and occupation (p<0.001) of the respondents were statistically significant to the change in lifestyle of the people (Table 3). In contrast to the impacts of ecotourism on the changing lifestyle of respondents, more than 83% of respondents think that ecotourism is responsible for creating economic inequality among locals in the study area (Table 4). No single socio-demographic characteristic was found to be significant. However, most respondents with low income and involved in agriculture think that ecotourism is the reason for economic inequality in the community (Table 4).

**Table 3:** Association between different socio-demographic characteristics and impacts of ecotourism in changing respondents’ lifestyles.

*Socio-demographic characteristics*	*Categories*	*Yes* *(count)*	*No* *(count)*	*Chi-square test (p-value)*
Ethnicity	BCN Janajati Madhesi Dalits	6 6 3 0	100 36 11 5	0.110
Gender	Male Female	5 10	80 72	0.153
Education	Illiterate Literate	2 13	42 110	0.230
Income	Low High	7 8	122 30	0.003
Occupation	Agriculture Business Others	3 12 0	133 4 15	0.000
Age	<25 years >25 years	5 10	9 143	0.000

**Table 4:** Association between different socio-demographic characteristics and impacts of ecotourism in causing economic inequality in the study area.

*Socio-demographic characteristics*	*Categories*	*Yes* *(count)*	*No* *(count)*	*Chi-square test (p-value)*
Ethnicity	BCN Janajati Madhesi Dalits	90 32 13 4	16 10 1 1	0.445
Gender	Male Female	68 71	17 11	0.254
Education	Illiterate Literate	38 101	6 22	0.517
Income	Low High	107 32	22 6	0.854
Occupation	Agriculture Business Others	114 11 14	22 5 1	0.170
Age	<25 years >25 years	3 25	11 128	0.625

More than 90% of respondents reported that ecotourism is not responsible for increasing commodity prices in the study area (Table 5). However, most respondents with high income reported that the increase in commodity prices in their place is due to ecotourism. The chi-square test shows that respondents’ ethnicity, income, occupation, and age significantly affect their response towards ecotourism’s impacts on an increase in commodity price (Table 5). Most respondents in agriculture occupations do not think that increased commodity price is due to ecotourism. Similarly, respondents with low income believe that there is no relationship between ecotourism and increased commodity prices (Table 5).

**Table 5:** Association between different socio-demographic characteristics and ecotourism as the reason for the increase in commodity price.

***Socio-demographic characteristics***	***Categories***	***Yes*** ***(count)***	***No*** ***(count)***	***Chi-square test (p-value)***
Ethnicity	BCN Janajati Madhesi Dalits	6 5 4 0	100 37 10 5	0.030
Gender	Male Female	6 9	79 73	0.376
Education	Illiterate Literate	2 13	42 110	0.230
Income	Low High	10 33	119 5	0.0305
Occupation	Agriculture Business Others	7 8 0	129 8 15	0.000
Age	<25 years >25 years	5 10	9 143	0.001

Most of the respondents (92%) perceived that infrastructure development in their area is due to ecotourism. They saw infrastructure development as the direct impact of ecotourism in their community. The chi-square test shows that only age and education are significant among the socio-demographic factors, with respondents’ perceptions of ecotourism impacts infrastructure development. More than 90% of the respondents who have at least school-level education think that infrastructure development in their area is due to ecotourism. Similarly, respondents of older age feel the same (Table 6).

**Table 6:** Association between different socio-demographic characteristics and respondents’ perceptions of the impacts of ecotourism in infrastructure development in their area.

*Socio-demographic characteristics*	*Categories*	*Improved* *(count)*	*No Change* *(count)*	*Chi-square test (p-value)*
Ethnicity	BCN Janajati Madhesi Dalits	99 39 12 4	7 3 2 1	0.553
Gender	Male Female	81 73	4 9	0.131
Education	Illiterate Literate	41 113	3 10	0.0380
Income	Low High	119 35	10 3	0.977
Occupation	Agriculture Business Others	125 14 15	11 2 0	0.410
Age	<25 years >25 years	11 143	3 10	0.046

### 3.3 Perceptions of local people on the social impacts of ecotourism

The results show that majority of the local people perceived that their knowledge base had been improved due to ecotourism. They believe that ecotourism impacts the educational development of their children. The result showed that most of the respondents with low income think that ecotourism activities in their area have helped to improve their children’s knowledge and language abilities (Table 7). The chi-square test shows the significant relationship between respondents’ income level and perceptions of ecotourism’s impacts on knowledge improvement (Table 7).

**Table 7:** Association between different socio-demographic characteristics and respondents’ perceptions towards the impacts of ecotourism on knowledge improvement of locals.

*Socio-demographic characteristics*	*Categories*	*Improved* *(count)*	*No change* *(count)*	*Chi-square test (p-value)*
Ethnicity	BCN Janajati Madhesi Dalits	76 33 10 4	30 9 4 1	0.832
Gender	Male Female	62 61	23 21	0.831
Education	Illiterate Literate	30 93	14 30	0.337
Income	Low High	91 32	38 6	0.052
Occupation	Agriculture Business Others	96 15 12	40 1 3	0.116
Age	<25 years >25 years	10 113	4 40	0.843

## 4 Discussion

Our study showed that local people from the Kumroj Buffer zone of Chitwan National Park have both positive and negative perceptions of the impacts of ecotourism. We found that people living around CNP are interested in ecotourism. Local people’s higher interest is obvious in areas like CNP, where the ecotourism industry is well established. As [41] suggested, the higher interest of local people in ecotourism is directly influenced by multiple factors, including local organizations and groups involved in providing ecotourism services. Such associations or networking agencies encourage local people to engage in ecotourism activities by conducting meetings, agri-business exhibitions, and extension programs. Our results also showed that the demographics and socioeconomic status of the local people were associated with their interest in ecotourism activities. This study revealed that most respondents recognized that infrastructure development in their area is due to increased ecotourism activities. Our results align with the existing studies by [42], ecotourism activities contribute to the infrastructure development of the area. We also found that old-aged and literate locals perceived ecotourism contributes to infrastructure development. The local people who perceive that their knowledge base is improved due to ecotourism activities are typically low-waged. People with low income appreciate the income from ecotourism as their primary source of income, which enables their children to get an education.

The study found that most residents were interested in ecotourism activities, and those interested residents were literate. This suggests that educated people are more interested in ecotourism. Educated people are an essential aspect of conserving natural resources and protected areas. These ecological protection areas also allow academic institutions to organize and implement field-based education for their students [43], which will enable them to see many benefits and positive aspects of ecotourism. This interest increase in ecotourism activities creates many different economic dynamics in the Kumroj Buffer Zone Area. A previous study suggests that due to the influx of tourists in the area, local people suffer from an increase in the price of commodities [1]. Still, local people from the Khumroj Buffer zone do not perceive that ecotourism significantly impacts commodity prices. This local people’s perception is significantly associated with their ethnicity, income, occupation, and age. In contrast, the study found that ecotourism is responsible for creating economic inequality among the local people. We feel that this aspect should be explored in-depth in future research.

Also, the study found that tourism helps in promoting infrastructure development. This perception of infrastructure development due to ecotourism activities is associated with the respondents’ age and education level. The previous study also showed that tourism activities encourage establishing facilities such as road access, hotel, lodges, resorts, restaurants, infrastructure, souvenir shops, grocery, and gift shops [12,44]. These facilities encourage large business personnel to engage in a range of tourism activities and create an opportunity to produce and sell local products such as vegetables, fruits, livestock, and handmade souvenirs [45]. In addition, ecotourism encourages small businesses such as nature guide services and travel and tours company in the areas. These infrastructure developments and other physical facilities enhance the economic benefits at the local, provincial and national levels. The economic benefits that residents perceive from ecotourism development include more employment opportunities, increased income, and business opportunities [1,9,19,30]. For instance, according to the CNP authorities, a total of 152,671 tourists visited and generated NRs 24,19,60,998.28 (US $1 = NRs 103) revenue in the fiscal year 2017/2018.

The social impacts of ecotourism on the local community are often perceived as improving residents’ quality of life, education standard (or literacy rate), leadership skills, and improvement in the language [1,46]. Due to the rise in income and economic activity, the local people can afford their basic needs, resulting in better living standards. Also, people can afford better schools for their children, be aware of their health, access to new technologies, and enhance social networks. Therefore, many locals switched from traditional cooking stoves to improved ones, reducing the dependency of forest products such as firewood. Our results align well with previous research where most of the residents switched to biogas plants and solar energy systems for the household energy source [47]. It is also found that CNP provides funds to the local people for road construction, school building maintenance, and biogas plants due to tourism activities [47]. Also, it creates opportunities for cultural exchange and the revitalization of local traditions [48]. Tourism can also result in social and cultural benefits such as more recreational opportunities for residents, improved public services and infrastructure, and a source of social change [1,48,49]. However, ecotourism may also have costs or adverse socio-cultural effects. As a source of change, ecotourism can have a negative impact on traditional family values, lead to cultural commercialization, and create socio-cultural conflicts in the host community due to differences in the economic welfare and purchasing power between the host community and tourists [50].

People perceived that ecotourism had played a vital role in the sustainable development of human welfare. A steady increase in tourists has positively and negatively impacted the region [51]. However, sustainable tourism management has always been a better strategy to mitigate tourism’s adverse effects [52]. The local peoples’ perceptions and attitudes towards ecotourism significantly contribute to sustainable tourism management.

## 5. Conclusions

Knowing local residents’ opinions is necessary in tourism destinations’ planning process and governance. Understanding their perceived impacts of ecotourism is needed for positive output. This study explored the relationship between local peoples’ perception of impacts and the importance of ecotourism with their socioeconomic and demographic characteristics. This study looked into the most overlooked aspect of ecotourism management, and the findings will serve as the foundation for local governments’ social aspects of ecotourism planning. Further, the study could serve as a guide to compare these famous ecotourism destinations with other tourist areas with similar characteristics, considering that results are not generalizable. The socio, economic and ecological conditions of each context influence the results; although they might have some common characteristics with other destinations, they are still unique to the particular local destination [17].

We hope this study will help fill in existing gaps in literature around the perceived impacts of ecotourism in underdeveloped countries Nepal. Though our research investigated the association between respondents’ socioeconomic and demographic characteristics and their perceived impacts and importance of ecotourism, we did not do any research that explored the causal effect of these associations. Furthermore, this study did not examine how local people perceived the ecological impacts of ecotourism. We suggest doing this as this will provide a more comprehensive understanding of the perceived impacts of ecotourism on all three aspects of sustainability viz, social, economic, and ecological.

## Supporting information

S1 File Survey guide used to collect data. (PDF)
