# Supplementary material for: Local people’s perception of the impacts and importance of ecotourism in Central Nepal
Source: PLoS One. 2022 May 27;17(5):e0268637. doi: 10.1371/journal.pone.0268637 (PMC9140242; doi:10.1371/journal.pone.0268637)
Supplement: S1 File — (PDF) [file pone.0268637.s001.pdf]

Survey Guide

1. General information about respondent:

Name:	Gender:
Age:	Caste:
Household size:	Education level:
Main source of income:	Monthly income:
Land for livelihood: Yes…..No….	Distance from forest:
No. of year you stay here:	

2. What recreational services are you currently getting from the forest?
   …….
3. Are you gaining economic benefits due to the inflow of tourist in KBZCF? Yes…..No…..
4. How economically benefitted are you from ecotourism? ………………………………………………………….
5. Is your any family member employed in ecotourism services in Kumroj? Yes……No……
6. If yes to Q. 5, how many members are employed there? ....
7. If no to Q. 5, why?
   ....
8. Have you established any enterprises to promote ecotourism? Yes……No…..
9. If yes to Q. 8, which services are you providing?
   a. …….
   b. …….
   c. …….
10. How many tourists visit you per day? ....
11. How much your family earn per day providing services for tourists? NRs……
12. If No to Q. 8, would you like to provide any services for tourists to gain economic returns? Yes…..No…..
13. If Yes to Q. 12, what is stopping you doing so?
    …….
14. What changes you notice in your daily life before five years and now due to ecotourism?

Variables	Before	After
Annual income of the family		
Economic status of family		
Intelligence of individual		
Infrastructures development		
Services and facilities		
Environmental services		

15. In your view, do you think tourism has any effect on the quality of environmental resources of Kumroj? Yes……No……

16. If Yes to Q. 6, is it positive or negative? ………….
17. If positive, what are the effects?
    a. …..
    b. …..
    c. …..
18. If negative, what are the effects?
    a. …..
    b. …..
    c. …..
19. In your opinion, does tourist flow increase in Kumroj beneficial to local people and their livelihood? Yes…..No…..
20. If Yes, How?
    a. Employment generation
    b. Additional income
    c. Business of local material
    d. Others (please specify)……………….
21. Who is getting more benefit from tourism activities?
    a. Park
    b. Business man
    c. Local people
    d. Others (Please specify)…………….
22. Is there any change in life style (especially expenditure pattern) due to earning from tourism? Yes…..No…..
23. Tourism has provided earning opportunity unequally among the villagers in Kumroj. This has increases economic inequality among villagers. Do you agree? Yes…..No…..Indifferent…..
24. Tourism may be responsible for increasing commodity prices in Kumroj compared to nearby villages. Villagers who sell their products to the tourists benefits from such increase in prices. Do you agree? Yes…..No…..Indifferent…..
25. Do you feel tourism has affected to nutrition of family members? i.e. selling more products and consuming less? Yes……No…..Indifferent…..
26. Are you satisfied with the budget allocated for community development by the park? Yes…..No…..
27. If No to Q. 26, what is your expectation regarding budget from the park? ………..
28. In your view what can be done for the sustainable management of ecotourism on Kumroj? ………..

Thank You
